# Assessment of competence of participants before and after 7-day intensive malaria microscopy training courses in Nigeria

**DOI:** 10.5281/zenodo.10870129

**Published:** 2015-06-09

**Authors:** Yetunde A. Olukosi, Chimere O. Agomo, Oluwagbemiga O. Aina, Samuel K. Akindele, Hilary I. Okoh, Margaret O. Akinyele, Olusola Ajibaye, Bassey A. Orok, Bamidele A. Iwalokun, Veronica Enya, Uche T. Igbasi, Samson Awolola

**Affiliations:** 1 Malaria Research Programme (MRP), Nigerian Institute of Medical Research (NIMR), 6 Edmond Crescent, Lagos, Nigeria

## Abstract

**Background:**

The accuracy of malaria diagnosis by microscopy has been a challenge in health facilities in Nigeria due to poor competence of microscopists and inability to report on malaria species other than *Plasmodium falciparum.* Short microscopy courses were conducted to improve the skills of laboratory personnel to perform malaria microscopy in public health facilities in Nigeria.

**Materials and Methods:**

Seven-day malaria microscopy courses were conducted annually between 2011 and 2013 for microscopists in public health facilities. The training courses contained theoretical and practical sessions. Impact of the training was evaluated by practical and theoretical pre- and post-training assessments on malaria slide reading, parasite enumeration and basic malariology.

**Results:**

The 102 participants who completed the training consisted of medical laboratory scientists (62; 60.8%), medical laboratory technicians (24; 23.5%) and other healthcare workers (16; 15.7%). The knowledge of basic malariology (theory) at pre- and post-tests were 34% (95% CI 31.7-36.3%) and 74.9% (95% CI 71.8-78.0%), respectively (P<0.001). The mean slide reading detection, species and counting agreements in pre-training assessment were 48.9%, 27.9% and 0%, respectively, and in post-training 56.8%, 39.2% and 25%, respectively. The mean species agreements in picture test pre- and post-training were 21.9% and 55.1%, respectively. There were significant differences (P<0.05) in the median pre-test scores in picture tests and basic malariology of the three categories of participants but not in malaria slide reading and parasite counting tests. However, post-training, a significant difference in test scores of the three categories of participants was recorded only for basic malariology (P=0.0003).

**Conclusions:**

The 7-day malaria microscopy courses significantly increased the knowledge and microscopy skills of the trainees and were sufficient to bridge the significant difference in baseline microscopy skills of the different categories of trainees that participated in the training courses.

## 1 Introduction

Accurate diagnosis and prompt effective treatment is central to effective malaria case management. Following the declaration for universal coverage of malaria control measures, most endemic countries, including Nigeria, adopted parasite-based diagnosis of malaria before treatment. To achieve this, malaria microscopy is recommended in tertiary and secondary health facilities where the capacity exists, whereas malaria rapid diagnostic tests (mRDTs) are recommended for use at the primary health facilities [1]. Nevertheless, in many primary healthcare centres malaria microscopy is the diagnostic method of choice because of the availability of laboratory personnel, microscopes and materials for preparing stained blood films.

The benefits of parasite-based diagnosis depend on accurate results and use of these results in malaria case management. Training and retraining of laboratory personnel involved in malaria diagnosis are central requirements to guaranteeing accurate and reliable malaria microscopy outcomes. Laboratory personnel need to be competent at detecting the presence of malaria parasites in blood samples and excluding them when absent even in the presence of artefacts. Unfortunately, inaccurate malaria microscopy is common in malaria-endemic countries, resulting in widespread over-diagnosis of malaria [2-4].

The factor by which malaria is over-diagnosed by diagnostic methods with low specificity is inversely related to the prevalence of malaria [5,6]. The implication is that when malaria prevalence falls, either with the scale up of intervention strategies like the use of artemisinin-based combination treatments (ACTs) and/or long-lasting insecticidal bednets (LLINs), or due to seasonal variation, the frequency with which malaria is over-diagnosed rises. It is therefore of utmost importance to build a competent cadre of laboratory personnel involved in malaria diagnosis in order to curtail malaria over-diagnosis, wastage of ACTs and improve patient care.

In a bid to contribute to improving the accuracy of malaria diagnosis in Nigeria, the Malaria Research Programme of the Nigerian Institute of Medical Research established a Malaria Microscopy Laboratory and has been involved in training of laboratory personnel. Here we discuss the performance of participants in a series of 7-day microscopy training courses over a three-year period.

## 2 Materials and Methods

The WHO prototype malaria microscopy course content [7] was adopted with minor modifications, including other malaria diagnostic options like rapid diagnostic tests and the Polymerase Chain Reaction (PCR). The participants were medical laboratory scientists and medical laboratory technicians from public health facilities providing routine malaria microscopy services, as well as other health professionals (lecturers, graduate students and researchers) involved in malaria research.

For the purpose of analysis, performances of participants were pooled from five 7-day training courses conducted over three years. The training course consisted of didactic lectures and hands-on sessions. Training modules included: (a) malaria epidemiology, diagnosis and treatment highlighting the different diagnostic methods for malaria and the changing pattern of malaria prevalence; (b) good laboratory practice, with emphasis on development and adherence to standard operating procedures; (c) malaria parasite quantification methods in thick and thin films; (d) morphological features of *Plasmodium falciparum*, *P. ovale*, *P. malaria* and *P. vivax*, highlighting distinguishing features; (e) preparation of blood films and Giemsa staining methods; (f) preparation of Giemsa stain (stock and working solutions); and (g) artefacts and pseudoparasites in stained blood films.

### 2.1 Training materials and tools

Microscopy training slide sets were prepared in accordance with the guidelines stated in the Malaria Microscopy Quality Assurance Manual [7]. The lectures were delivered through the use of audio-visual aid (power-point presentations and videos) employing adult learning techniques. Most of the lecture series and illustrations were adapted from malaria microscopy training power-point slides obtained from Walter Reed Army Research Institute, Kisumu, Kenya. Participants were provided with current copies of WHO malaria microscopy learner’s guide and bench aids. The training slides were prepared from field samples and validated by a panel of three expert malaria microscopists for parasite species, stage and count. Quality control for the slides was by double reading of the slides blindly by two microscopists and the mean of parasite counts within 20% discrepancy was taken.

### 2.2 Performance evaluation

The participants were assessed on the first day before training commenced and on the seventh day after completing the training modules. The areas of assessment were malaria parasite detection and species identification (pictures and hands-on); parasite quantitation (hands-on); general knowledge, with a focus on basic malariology and good clinical and laboratory practice (multiple-choice questions). The assessment criteria for pictures and slide reading were defined as follows:

$$\text{Detection agreement (DA) =}\frac{Number\;of\;true\;positive+Number\;of\;true\;negative}{Total\;number\;of\;slides\;exa\min ed}\times 100$$

$$\text{Species agreement (SpA) }=\frac{Number\;of\;correct\;parasite\;species\;identification}{Total\;number\;of\;positive\;slides\;exa\min ed}\times 100$$

$$\text{Stage agreement (StA)}=\frac{Number\;of\;correct\;parasite\;stage\;identification}{Total\;number\;of\;positive\;slides\;exa\min ed}\times 100$$

$$\text{Counting agreement (CA)}=\frac{Number\;of\;counts\;within\pm 20\%\;of\;reference\;count}{Total\;number\;of\;slides\;counted}\times 100$$

$$\text{Percentage improvement}=\frac{Post\;test\;score-pretest\;score}{\mathrm{Pr}etest\;score}\times 100$$

### 2.3 Data management and statistical analysis

All data were entered in a database using Microsoft Office Excel 2010 and analysed with EPI INFO 3.5.4. The performance scores for detection of parasitaemia, parasite species and stage were rated as ‘good’ (≥70%), ‘fair’ (50-69%) and ‘poor’ (<50%), except for counting agreement. The rating for counting agreement was: ‘good’ ≥ 30%; ‘fair’ = 20-29% and ‘poor’ <20%. The ‘good’ rating is in accordance with minimum score for a level-3 microscopist as contained in the WHO accreditation scheme for microscopists [7]. The results of the tests were analysed by Wilcoxon test. A *P*-value <0.05 was considered statistically significant.

## 3 Results

A total of 102 performance records of participants who completed all the modules of the 7-day malaria microscopy capacity building workshops conducted between 2011 and 2013 were analysed. Participants were from Lagos state (73, 71.6%), Ogun State (21, 20.6%) and other states (8, 7.8%). Over half of the participants (62, 60.8%) were medical laboratory scientists (MLSs), 24 (23.5%) were medical laboratory technicians (MLTs), whereas 16 others (15.7%) were research scientists, graduate students and clinicians. The male to female ratio was 1:1.8.

**Figure 1.**
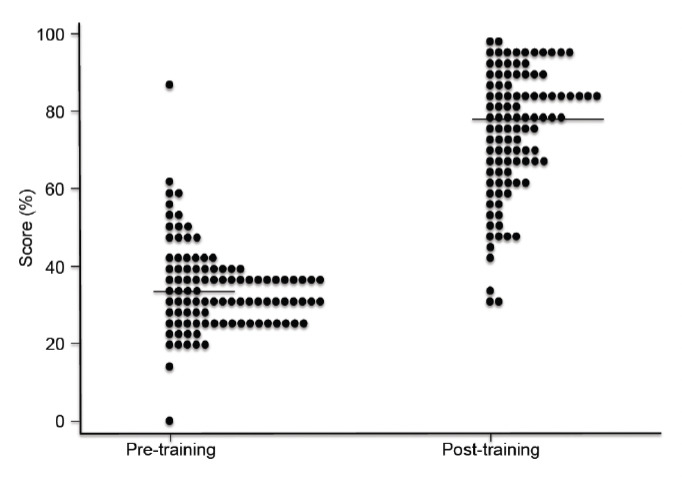
Dot plot of scores (multiple choice questions) of participants in basic malariology (symbols represent the score of an individual participant; the lines represent the median scores) pre-and post training.

Overall, the pre- and post-training mean scores in basic malariology were 34% and 74.9%, respectively (Table 1). The distribution of the scores is shown in Figure 1. The mean score of slide reading DA, SpA and CA in pre-training assessment were 48.9%, 27.9% and 0%, respectively, while the levels in post-training were 56.8%, 39.2% and 25%, respectively. The mean scores for DA and SA in picture test pre-training were 58% and 22.4%, respectively, and 87.8% and 55.3% at post-training, respectively.

**Table 1.** Overall mean performance of the different cadres of participants before and after a 7-day course on malaria microscopy.

Test type	Assessment	Pre-training	Post-training	*P*-value
Theory	Basic malariology (95% CI)	34.0% (31.7 - 36.3%)	74.9 (71.8 - 78.0%)	<0.001
Picture	DA_Pic (95% CI)	58% (52.7 - 63.3%)	87.8 (85.1 - 90.6%)	<0.001
	SpA_Pic (95% CI)	22.4% (19.1 - 25.8%)	55.3% (52.1 - 58.5%)	<0.001
	StA_Pic (95% CI)	29.3% (25.7 - 33.0%)	65.8% (62.6 - 69.0%)	<0.001
Slide reading	DA_slide (95% CI)	48.9% (45.2 - 52.6%)	56.8% (54.5 - 59.0%)	<0.001
	SpA_Slide (95% CI)	27.9% (24.0 - 31.7%)	39.2% (35.2 - 43.1%)	<0.001
	StA_Slide (95% CI)	29.1% (25.8 - 32.4%)	46.6% (43.5 - 49.7%)	<0.001
	Counting (95% CI)	NA	25% (21.7 - 28.3%)	<0.001

DA = Detection agreement; SpA = Species agreement; StA = Stage agreement; NA = not applicable.

There were significant differences (*P*<0.05) in the median pre-test scores in theoretical tests (picture and basic malariology) of the three categories of participants, but their median scores in the practical tests were similar (*P*>0.05). However, at post-test, there was no significant difference in median scores of the three categories of participants, except for basic malariology (*P*=0.0003).

The trend of improvement was similar across the parameters for the different cadres of trainees, although significant differences in the median percentage improvements of the three categories of trainees were recorded in basic malariology (*P*=0.02), picture parasite detection (picture) (*P*=0.03) and stage identification (slide) (*P*=0.018) (Table 2).

**Table 2.** Comparison of scores performance (%) of the three cadres of participants pre- and post-training.

Category		Basic malariology	Picture			Slide			Parasite Count
			DA	SpA	StA	DA	SpA	StA	
Pre-test	MLS*	29.6	65.0	21.9	32.8	50.0	30.0	30.0	-
	MLT	35.2	53.3	16.1	18.0	50.0	20.0	16.7	-
	Others	41.5	76.7	35.9	32.0	45.0	30.0	25.8	-
	P value	**<0.001**	**0.005**	**0.001**	**0.012**	0.20	0.13	**0.005**	
Post-test	MLS	77.8	90.0	59.5	68.6	57.5	38.1	44.0	20.0
	MLT	70.4	88.8	49.3	60.3	55.0	30.0	41.3	20.0
	Others	92.3	87.5	61.3	73.8	61.3	40.0	50.6	28.3
	P value	**<0.001**	0.65	0.14	**0.05**	0.07	0.09	0.47	0.17
% Improvement	MLS	147.6	31.2	137.8	96.7	11.1	33.3	23.5	
	MLT	90.9	45.8	143.8	90.2	10.0	20.0	178.3	
	Others	109.4	17.7	54.0	111.1	33.3	50.0	77.9	
	P value	**0.002**	**0.03**	0.06	0.53	0.34	0.90	**0.018**	

* MLS = Medical laboratory scientist (*n*=62); MLT = Medical laboratory technician (*n*=24); Others (*n*=16).

**Figure 2.**
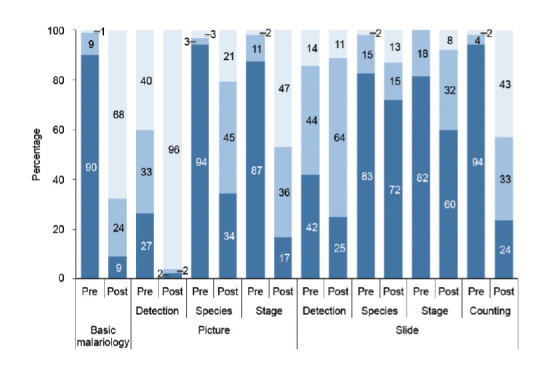
Overall performance of participants in malaria microscopy pre- and post training. Score rating for all assessments except counting: Poor (dark blue)= <50%; Fair (light blue) = 50-69%; Good (grey) = ≥70%). For counting the values are Poor = <20%; Fair = 20-29%; Good = ≥30%).

**Figure 3.**
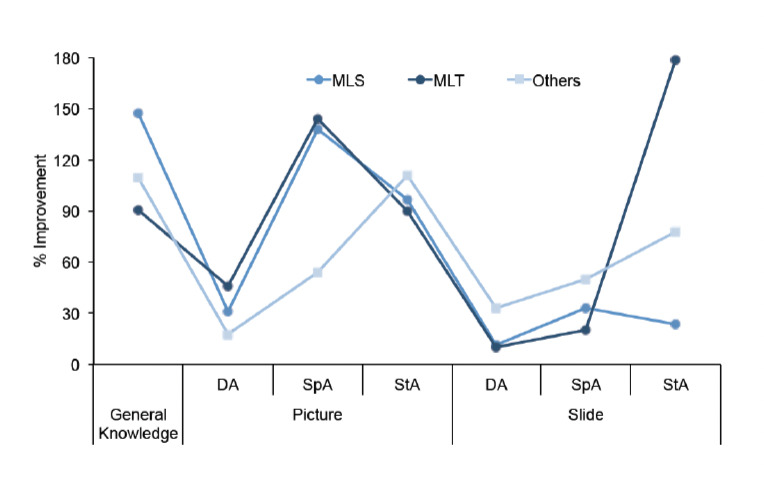
Improvement (in %) for the different assessment areas according to trainee groups (MLS = medical laboratory scientists; MLT = medical laboratory technicians).

MLSs had the highest percentage improvement in basic malariology (147.6%); MLTs had the highest percentage improvement for picture detection (45.8%), picture species (143.8%) and slide stage (178.3%); whereas others had the highest percentage improvement in picture stage (111.1%), slide detection (33.3%) and slide species (50%) (Fig. 2). The ‘poor’ ratings at post-test were reduced for all parameters, whereas the ‘fair’ and ‘good’ ratings increased compared to the pre-tests (Fig. 3).

## 4 Discussion

By implementing the 7-day malaria microscopy training courses, the post-test scores of the participants were observed to be significantly higher than their pre-test scores. These findings indicate that knowledge and skills were acquired during the training courses. Most of the trainees could not count parasites at baseline but were able to do so at the end of the trainings, with over 40% of them counting well. Similar post training improvements were observed in a training evaluation in Uganda [8].

The large differences observed in the test performance of the participants rated as ‘good’ in slide versus picture assessments might be attributed to the technical difficulty in manipulating the microscope to find and recognise malaria parasites in stained slides within the given time allocated for slide examination. Slide reading assessments are better than parasite picture assessments in ascertaining trainee’s level of microscopy proficiency as they simulate field conditions more realistically and are the criteria for WHO competency assessment of microscopists [7]. The use of parasite pictures, however, complements the overall malaria microscopy learning process.

A modest improvement in the mean DA in slide reading was observed in these trainings. This was also seen in the reduction in proportion of participants with ‘poor’ rating in detection agreement in slide reading. Increase in detection agreement will result in reduced misdiagnosis of malaria and hence a reduction of unnecessary use of ACTs [9, 10].

Length of schooling and previous level of exposure and involvement in malaria microscopy are criteria that help to determine the length of training required to acquire necessary competency levels [7]. The participants of these trainings had at least 12 years of formal education. Laboratory personnel [MLTs (mid-level cadre) and MLSs (high-level cadre)] routinely provide malaria microscopy services in primary and secondary health facilities, respectively. The other groups [clinicians and researchers (high-level cadres)] are mostly involved in research studies with a malaria microscopy component.

Generally, the other participants had the highest scores at post-test followed by MLS. However, the differences in scores were not significant, except in basic malariology. This shows that even low level cadre laboratory personnel are trainable in malaria microscopy and have the potential to become very good microscopists if given the opportunity to attend regular re-training courses and are involved in external quality assurance programmes [11-13]. Access to regular training and refresher courses is a challenge considering the few organisations providing such service in a big country like Nigeria. Attention has been focused on training MLS cadres in public secondary health facilities. The MLT cadres have been excluded because they are expected to use malaria rapid diagnostic tests but they also carry out malaria microscopy in primary healthcare facilities and even believe their results are better than mRDT results (Mokuolu *et al.*, 2013; unpublished report).

## 5 Conclusions

The 7-day malaria microscopy courses significantly increased the knowledge and microscopy skills of the trainees and were sufficient to bridge the significant difference in baseline microscopy skills of the different categories of trainees that participated in the training courses.
